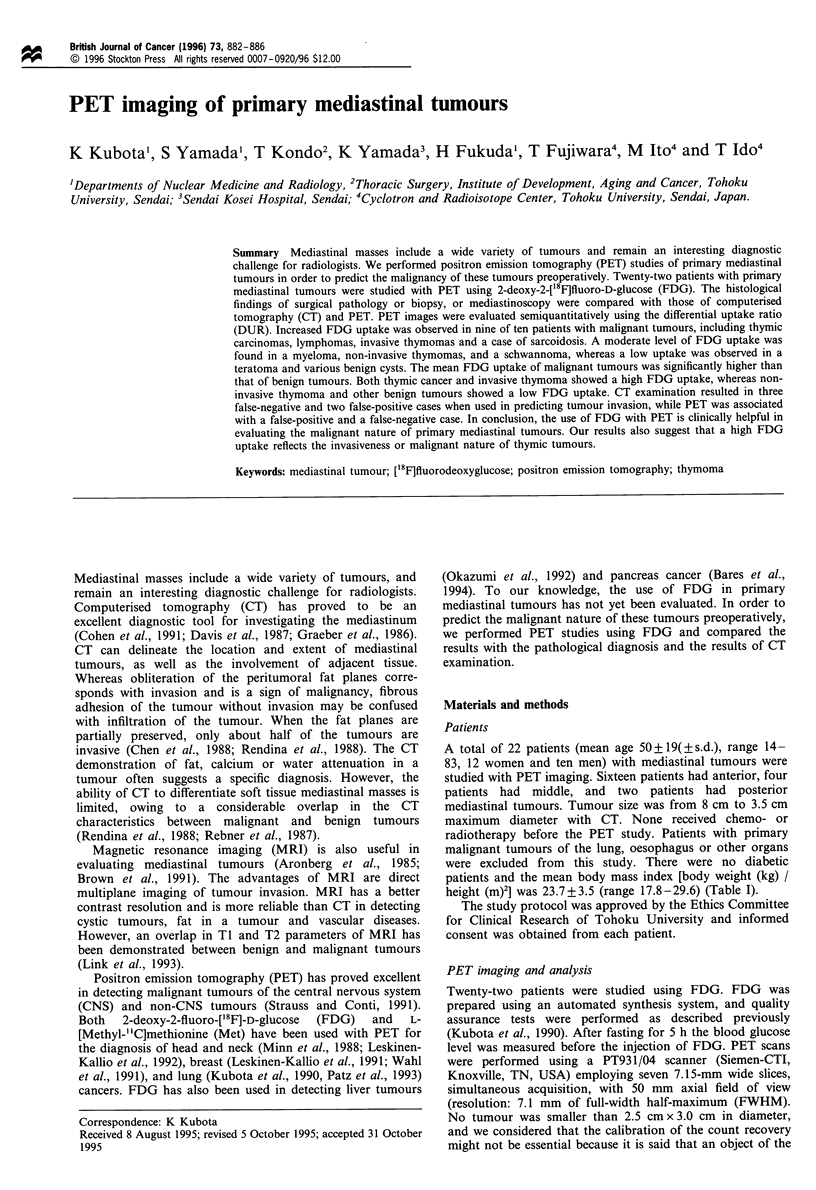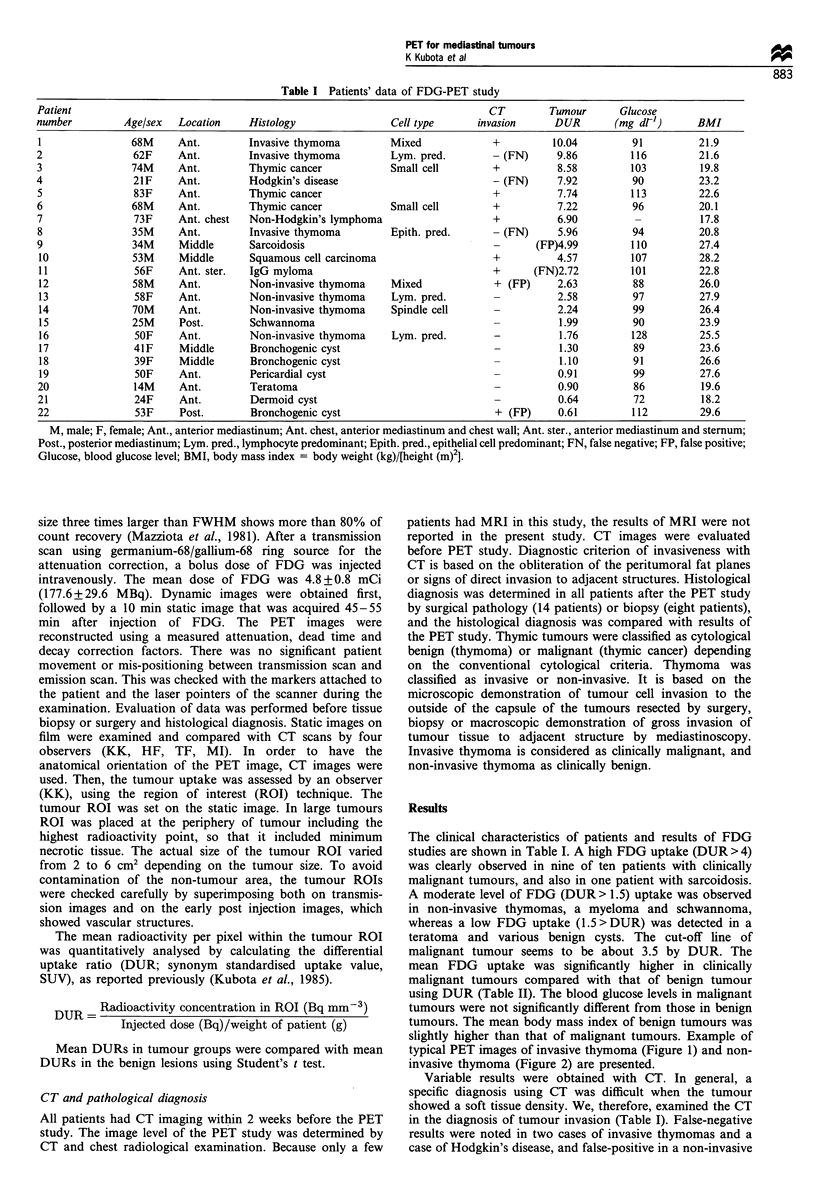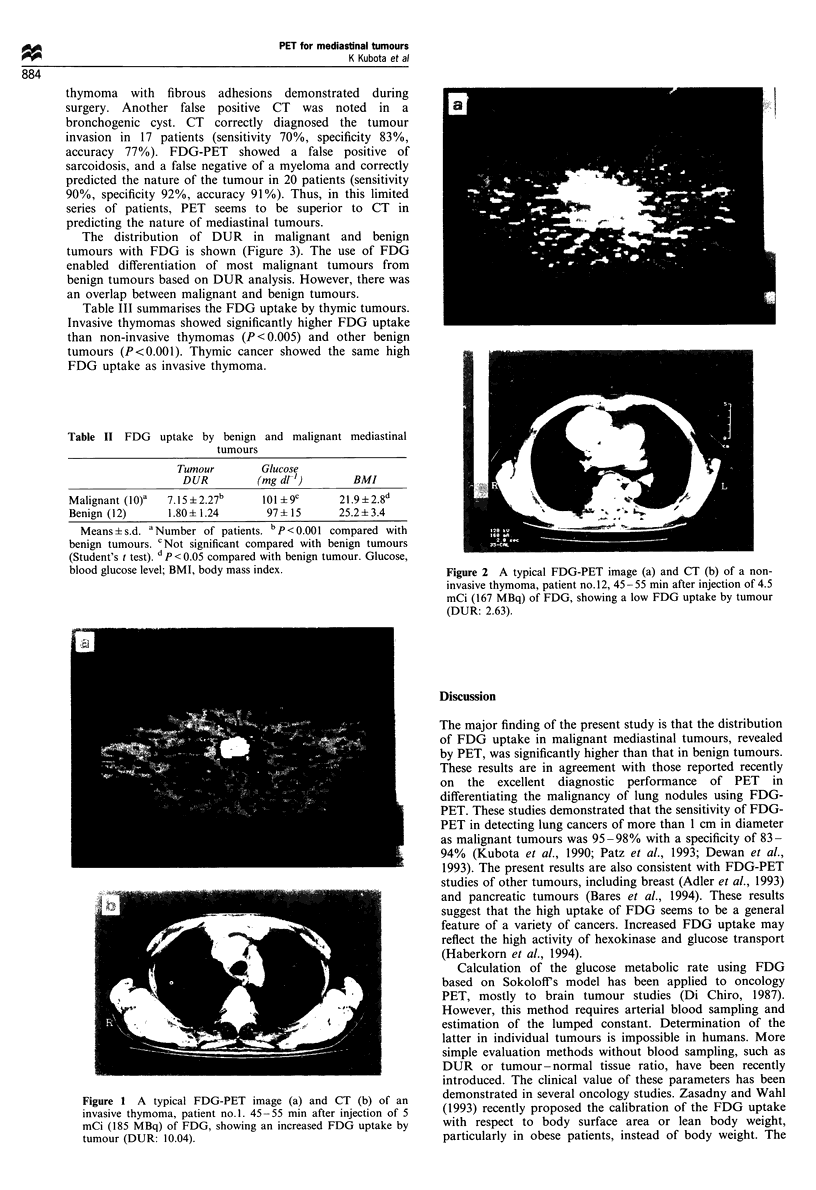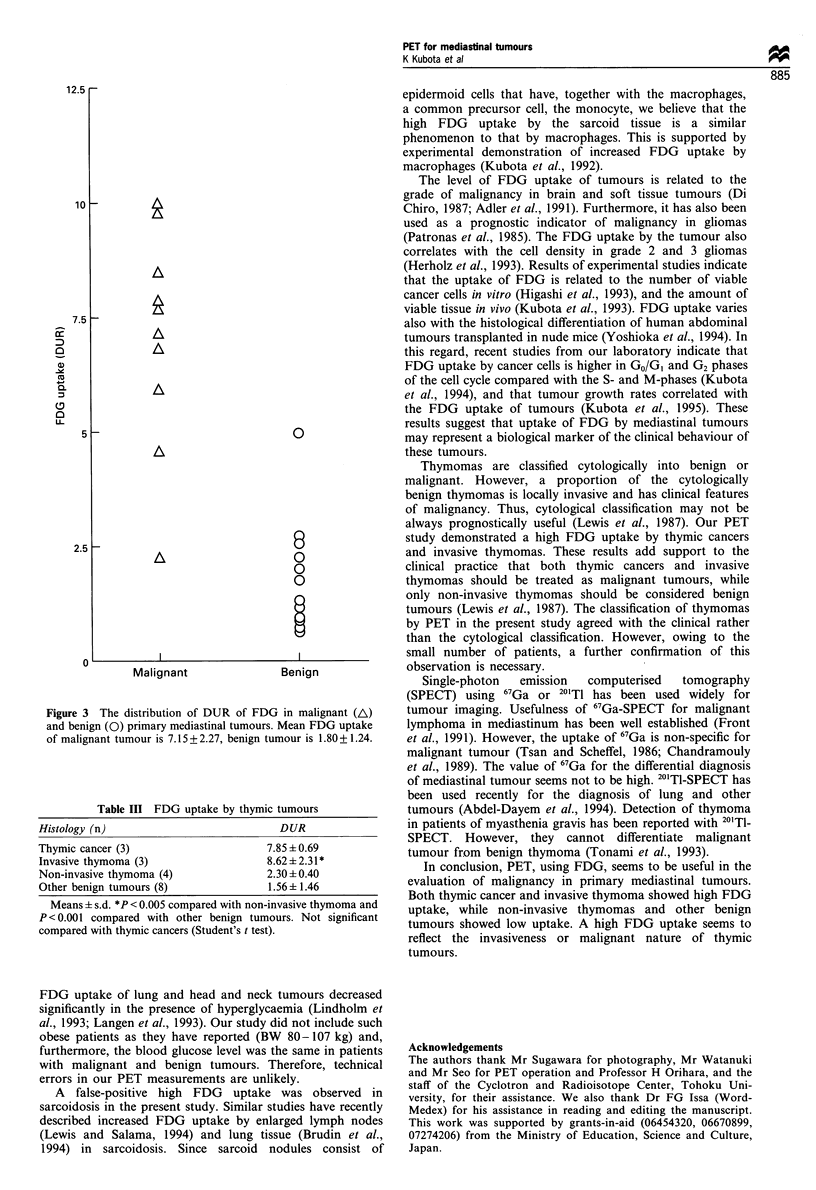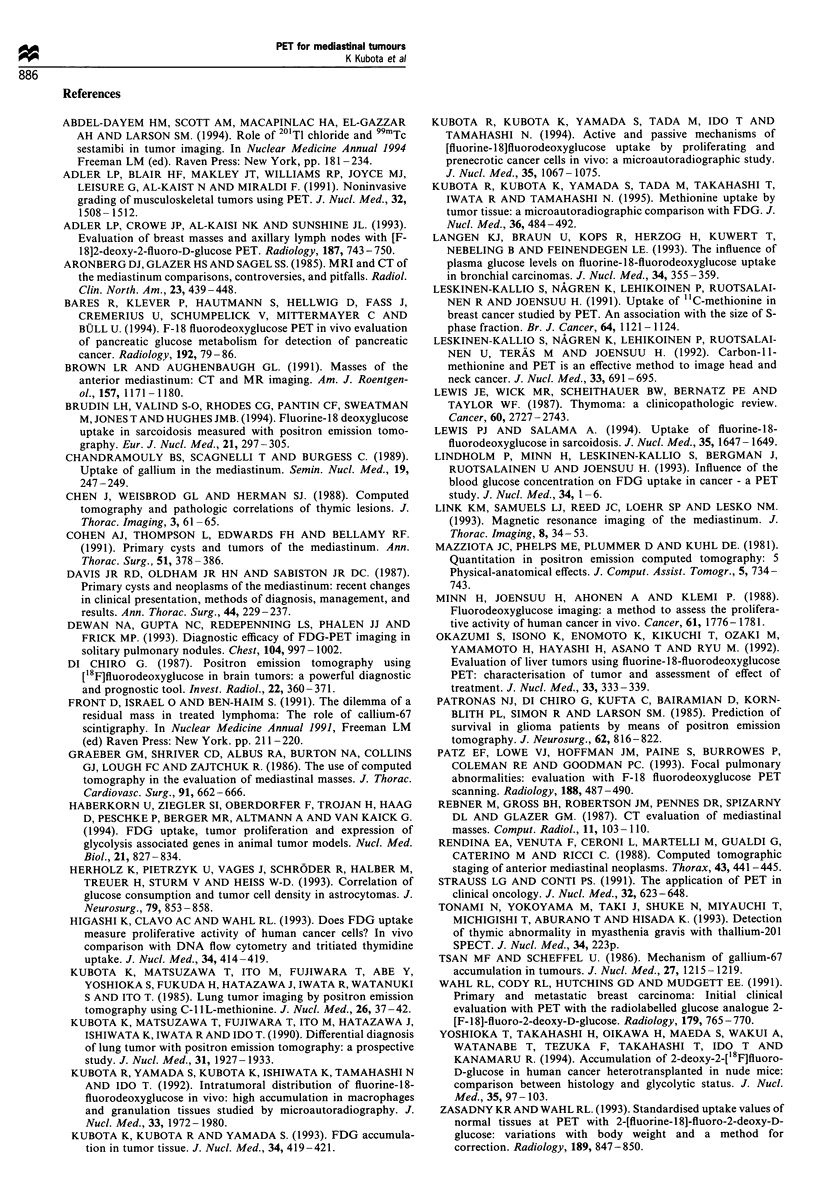# PET imaging of primary mediastinal tumours.

**DOI:** 10.1038/bjc.1996.157

**Published:** 1996-04

**Authors:** K. Kubota, S. Yamada, T. Kondo, K. Yamada, H. Fukuda, T. Fujiwara, M. Ito, T. Ido

**Affiliations:** Department of Nuclear Medicine and Radiology, Tohoku University, Sendai, Japan.

## Abstract

**Images:**


British Journal of Cancer (1996) 73, 882-886
© 1996 Stockton Press All rights reserved 0007-0920/96 $12.00

### PET imaging of primary mediastinal tumours

K Kubota[1], S Yamada[1], T Kondo[2], K Yamada[3], H Fukuda[1], T Fujiwara[4], M Ito[4] and T Ido[4]

[1]*Departments of Nuclear Medicine and Radiology,* [2]*Thoracic Surgery, Institute of Development, Aging and Cancer, Tohoku University, Sendai;* [3]*Sendai Kosei Hospital, Sendai;* [4]*Cyclotron and Radioisotope Center, Tohoku University, Sendai, Japan.*

**Summary** Mediastinal masses include a wide variety of tumours and remain an interesting diagnostic challenge for radiologists. We performed positron emission tomography (PET) studies of primary mediastinal tumours in order to predict the malignancy of these tumours preoperatively. Twenty-two patients with primary mediastinal tumours were studied with PET using 2-deoxy-2-[$^{18}$F]fluoro-D-glucose (FDG). The histological findings of surgical pathology or biopsy, or mediastinoscopy were compared with those of computerised tomography (CT) and PET. PET images were evaluated semiquantitatively using the differential uptake ratio (DUR). Increased FDG uptake was observed in nine of ten patients with malignant tumours, including thymic carcinomas, lymphomas, invasive thymomas and a case of sarcoidosis. A moderate level of FDG uptake was found in a myeloma, non-invasive thymomas, and a schwannoma, whereas a low uptake was observed in a teratoma and various benign cysts. The mean FDG uptake of malignant tumours was significantly higher than that of benign tumours. Both thymic cancer and invasive thymoma showed a high FDG uptake, whereas non-invasive thymoma and other benign tumours showed a low FDG uptake. CT examination resulted in three false-negative and two false-positive cases when used in predicting tumour invasion, while PET was associated with a false-positive and a false-negative case. In conclusion, the use of FDG with PET is clinically helpful in evaluating the malignant nature of primary mediastinal tumours. Our results also suggest that a high FDG uptake reflects the invasiveness or malignant nature of thymic tumours.

**Keywords:** mediastinal tumour; [$^{18}$F]fluorodeoxyglucose; positron emission tomography; thymoma

Mediastinal masses include a wide variety of tumours, and remain an interesting diagnostic challenge for radiologists. Computerised tomography (CT) has proved to be an excellent diagnostic tool for investigating the mediastinum (Cohen *et al.*, 1991; Davis *et al.*, 1987; Graeber *et al.*, 1986). CT can delineate the location and extent of mediastinal tumours, as well as the involvement of adjacent tissue. Whereas obliteration of the peritumoral fat planes corresponds with invasion and is a sign of malignancy, fibrous adhesion of the tumour without invasion may be confused with infiltration of the tumour. When the fat planes are partially preserved, only about half of the tumours are invasive (Chen *et al.*, 1988; Rendina *et al.*, 1988). The CT demonstration of fat, calcium or water attenuation in a tumour often suggests a specific diagnosis. However, the ability of CT to differentiate soft tissue mediastinal masses is limited, owing to a considerable overlap in the CT characteristics between malignant and benign tumours (Rendina *et al.*, 1988; Rebner *et al.*, 1987).

Magnetic resonance imaging (MRI) is also useful in evaluating mediastinal tumours (Aronberg *et al.*, 1985; Brown *et al.*, 1991). The advantages of MRI are direct multiplane imaging of tumour invasion. MRI has a better contrast resolution and is more reliable than CT in detecting cystic tumours, fat in a tumour and vascular diseases. However, an overlap in T1 and T2 parameters of MRI has been demonstrated between benign and malignant tumours (Link *et al.*, 1993).

Positron emission tomography (PET) has proved excellent in detecting malignant tumours of the central nervous system (CNS) and non-CNS tumours (Strauss and Conti, 1991). Both 2-deoxy-2-fluoro-[$^{18}$F]-D-glucose (FDG) and L-[Methyl-$^{11}$C]methionine (Met) have been used with PET for the diagnosis of head and neck (Minn *et al.*, 1988; Leskinen-Kallio *et al.*, 1992), breast (Leskinen-Kallio *et al.*, 1991; Wahl *et al.*, 1991), and lung (Kubota *et al.*, 1990, Patz *et al.*, 1993) cancers. FDG has also been used in detecting liver tumours (Okazumi *et al.*, 1992) and pancreas cancer (Bares *et al.*, 1994). To our knowledge, the use of FDG in primary mediastinal tumours has not yet been evaluated. In order to predict the malignant nature of these tumours preoperatively, we performed PET studies using FDG and compared the results with the pathological diagnosis and the results of CT examination.

### Materials and methods

### *Patients*

A total of 22 patients (mean age $50 \pm 19(\pm$s.d.), range 14–83, 12 women and ten men) with mediastinal tumours were studied with PET imaging. Sixteen patients had anterior, four patients had middle, and two patients had posterior mediastinal tumours. Tumour size was from 8 cm to 3.5 cm maximum diameter with CT. None received chemo- or radiotherapy before the PET study. Patients with primary malignant tumours of the lung, oesophagus or other organs were excluded from this study. There were no diabetic patients and the mean body mass index [body weight (kg) / height (m)$^2$] was $23.7 \pm 3.5$ (range 17.8–29.6) (Table I).

The study protocol was approved by the Ethics Committee for Clinical Research of Tohoku University and informed consent was obtained from each patient.

### *PET imaging and analysis*

Twenty-two patients were studied using FDG. FDG was prepared using an automated synthesis system, and quality assurance tests were performed as described previously (Kubota *et al.*, 1990). After fasting for 5 h the blood glucose level was measured before the injection of FDG. PET scans were performed using a PT931/04 scanner (Siemen-CTI, Knoxville, TN, USA) employing seven 7.15-mm wide slices, simultaneous acquisition, with 50 mm axial field of view (resolution: 7.1 mm of full-width half-maximum (FWHM). No tumour was smaller than 2.5 cm × 3.0 cm in diameter, and we considered that the calibration of the count recovery might not be essential because it is said that an object of the

Correspondence: K Kubota
Received 8 August 1995; revised 5 October 1995; accepted 31 October 1995

Table I Patients' data of FDG-PET study

| *Patient number* | *Age/sex* | *Location* | *Histology* | *Cell type* | *CT invasion* | *Tumour DUR* | *Glucose (mg $dl^{-1}$)* | *BMI* |
|---|---|---|---|---|---|---|---|---|
| 1 | 68M | Ant. | Invasive thymoma | Mixed | + | 10.04 | 91 | 21.9 |
| 2 | 62F | Ant. | Invasive thymoma | Lym. pred. | – (FN) | 9.86 | 116 | 21.6 |
| 3 | 74M | Ant. | Thymic cancer | Small cell | + | 8.58 | 103 | 19.8 |
| 4 | 21F | Ant. | Hodgkin's disease | | – (FN) | 7.92 | 90 | 23.2 |
| 5 | 83F | Ant. | Thymic cancer | | + | 7.74 | 113 | 22.6 |
| 6 | 68M | Ant. | Thymic cancer | Small cell | + | 7.22 | 96 | 20.1 |
| 7 | 73F | Ant. chest | Non-Hodgkin's lymphoma | | + | 6.90 | – | 17.8 |
| 8 | 35M | Ant. | Invasive thymoma | Epith. pred. | – (FN) | 5.96 | 94 | 20.8 |
| 9 | 34M | Middle | Sarcoidosis | | – | (FP)4.99 | 110 | 27.4 |
| 10 | 53M | Middle | Squamous cell carcinoma | | + | 4.57 | 107 | 28.2 |
| 11 | 56F | Ant. ster. | IgG myloma | | + | (FN)2.72 | 101 | 22.8 |
| 12 | 58M | Ant. | Non-invasive thymoma | Mixed | + (FP) | 2.63 | 88 | 26.0 |
| 13 | 58F | Ant. | Non-invasive thymoma | Lym. pred. | – | 2.58 | 97 | 27.9 |
| 14 | 70M | Ant. | Non-invasive thymoma | Spindle cell | – | 2.24 | 99 | 26.4 |
| 15 | 25M | Post. | Schwannoma | | – | 1.99 | 90 | 23.9 |
| 16 | 50F | Ant. | Non-invasive thymoma | Lym. pred. | – | 1.76 | 128 | 25.5 |
| 17 | 41F | Middle | Bronchogenic cyst | | – | 1.30 | 89 | 23.6 |
| 18 | 39F | Middle | Bronchogenic cyst | | – | 1.10 | 91 | 26.6 |
| 19 | 50F | Ant. | Pericardial cyst | | – | 0.91 | 99 | 27.6 |
| 20 | 14M | Ant. | Teratoma | | – | 0.90 | 86 | 19.6 |
| 21 | 24F | Ant. | Dermoid cyst | | – | 0.64 | 72 | 18.2 |
| 22 | 53F | Post. | Bronchogenic cyst | | + (FP) | 0.61 | 112 | 29.6 |

M, male; F, female; Ant., anterior mediastinum; Ant. chest, anterior mediastinum and chest wall; Ant. ster., anterior mediastinum and sternum; Post., posterior mediastinum; Lym. pred., lymphocyte predominant; Epith. pred., epithelial cell predominant; FN, false negative; FP, false positive; Glucose, blood glucose level; BMI, body mass index = body weight (kg)/[height (m)$^2$].

size three times larger than FWHM shows more than 80% of count recovery (Mazziota *et al.*, 1981). After a transmission scan using germanium-68/gallium-68 ring source for the attenuation correction, a bolus dose of FDG was injected intravenously. The mean dose of FDG was $4.8 \pm 0.8$ mCi ($177.6 \pm 29.6$ MBq). Dynamic images were obtained first, followed by a 10 min static image that was acquired 45–55 min after injection of FDG. The PET images were reconstructed using a measured attenuation, dead time and decay correction factors. There was no significant patient movement or mis-positioning between transmission scan and emission scan. This was checked with the markers attached to the patient and the laser pointers of the scanner during the examination. Evaluation of data was performed before tissue biopsy or surgery and histological diagnosis. Static images on film were examined and compared with CT scans by four observers (KK, HF, TF, MI). In order to have the anatomical orientation of the PET image, CT images were used. Then, the tumour uptake was assessed by an observer (KK), using the region of interest (ROI) technique. The tumour ROI was set on the static image. In large tumours ROI was placed at the periphery of tumour including the highest radioactivity point, so that it included minimum necrotic tissue. The actual size of the tumour ROI varied from 2 to 6 cm$^2$ depending on the tumour size. To avoid contamination of the non-tumour area, the tumour ROIs were checked carefully by superimposing both on transmission images and on the early post injection images, which showed vascular structures.

The mean radioactivity per pixel within the tumour ROI was quantitatively analysed by calculating the differential uptake ratio (DUR; synonym standardised uptake value, SUV), as reported previously (Kubota *et al.*, 1985).

$$\mathrm{DUR} = \frac{\text{Radioactivity concentration in ROI (Bq mm}^{-3}\text{)}}{\text{Injected dose (Bq)/weight of patient (g)}}$$

Mean DURs in tumour groups were compared with mean DURs in the benign lesions using Student's *t* test.

### *CT and pathological diagnosis*

All patients had CT imaging within 2 weeks before the PET study. The image level of the PET study was determined by CT and chest radiological examination. Because only a few patients had MRI in this study, the results of MRI were not reported in the present study. CT images were evaluated before PET study. Diagnostic criterion of invasiveness with CT is based on the obliteration of the peritumoral fat planes or signs of direct invasion to adjacent structures. Histological diagnosis was determined in all patients after the PET study by surgical pathology (14 patients) or biopsy (eight patients), and the histological diagnosis was compared with results of the PET study. Thymic tumours were classified as cytological benign (thymoma) or malignant (thymic cancer) depending on the conventional cytological criteria. Thymoma was classified as invasive or non-invasive. It is based on the microscopic demonstration of tumour cell invasion to the outside of the capsule of the tumours resected by surgery, biopsy or macroscopic demonstration of gross invasion of tumour tissue to adjacent structure by mediastinoscopy. Invasive thymoma is considered as clinically malignant, and non-invasive thymoma as clinically benign.

### Results

The clinical characteristics of patients and results of FDG studies are shown in Table I. A high FDG uptake (DUR > 4) was clearly observed in nine of ten patients with clinically malignant tumours, and also in one patient with sarcoidosis. A moderate level of FDG (DUR > 1.5) uptake was observed in non-invasive thymomas, a myeloma and schwannoma, whereas a low FDG uptake (1.5 > DUR) was detected in a teratoma and various benign cysts. The cut-off line of malignant tumour seems to be about 3.5 by DUR. The mean FDG uptake was significantly higher in clinically malignant tumours compared with that of benign tumour using DUR (Table II). The blood glucose levels in malignant tumours were not significantly different from those in benign tumours. The mean body mass index of benign tumours was slightly higher than that of malignant tumours. Example of typical PET images of invasive thymoma (Figure 1) and non-invasive thymoma (Figure 2) are presented.

Variable results were obtained with CT. In general, a specific diagnosis using CT was difficult when the tumour showed a soft tissue density. We, therefore, examined the CT in the diagnosis of tumour invasion (Table I). False-negative results were noted in two cases of invasive thymomas and a case of Hodgkin's disease, and false-positive in a non-invasive thymoma with fibrous adhesions demonstrated during surgery. Another false positive CT was noted in a bronchogenic cyst. CT correctly diagnosed the tumour invasion in 17 patients (sensitivity 70%, specificity 83%, accuracy 77%). FDG-PET showed a false positive of sarcoidosis, and a false negative of a myeloma and correctly predicted the nature of the tumour in 20 patients (sensitivity 90%, specificity 92%, accuracy 91%). Thus, in this limited series of patients, PET seems to be superior to CT in predicting the nature of mediastinal tumours.

The distribution of DUR in malignant and benign tumours with FDG is shown (Figure 3). The use of FDG enabled differentiation of most malignant tumours from benign tumours based on DUR analysis. However, there was an overlap between malignant and benign tumours.

Table III summarises the FDG uptake by thymic tumours. Invasive thymomas showed significantly higher FDG uptake than non-invasive thymomas ($P<0.005$) and other benign tumours ($P<0.001$). Thymic cancer showed the same high FDG uptake as invasive thymoma.

**Table II** FDG uptake by benign and malignant mediastinal tumours

| | *Tumour DUR* | *Glucose (mg dl$^{-1}$)* | *BMI* |
|---|---|---|---|
| Malignant (10)[a] | 7.15 ± 2.27[b] | 101 ± 9[c] | 21.9 ± 2.8[d] |
| Benign (12) | 1.80 ± 1.24 | 97 ± 15 | 25.2 ± 3.4 |

Means ± s.d. [a]Number of patients. [b]$P<0.001$ compared with benign tumours. [c]Not significant compared with benign tumours (Student's *t* test). [d]$P<0.05$ compared with benign tumour. Glucose, blood glucose level; BMI, body mass index.

**Figure 1** A typical FDG-PET image (a) and CT (b) of an invasive thymoma, patient no.1. 45–55 min after injection of 5 mCi (185 MBq) of FDG, showing an increased FDG uptake by tumour (DUR: 10.04).

**Figure 2** A typical FDG-PET image (a) and CT (b) of a non-invasive thymoma, patient no.12, 45–55 min after injection of 4.5 mCi (167 MBq) of FDG, showing a low FDG uptake by tumour (DUR: 2.63).

### Discussion

The major finding of the present study is that the distribution of FDG uptake in malignant mediastinal tumours, revealed by PET, was significantly higher than that in benign tumours. These results are in agreement with those reported recently on the excellent diagnostic performance of PET in differentiating the malignancy of lung nodules using FDG-PET. These studies demonstrated that the sensitivity of FDG-PET in detecting lung cancers of more than 1 cm in diameter as malignant tumours was 95–98% with a specificity of 83–94% (Kubota *et al.*, 1990; Patz *et al.*, 1993; Dewan *et al.*, 1993). The present results are also consistent with FDG-PET studies of other tumours, including breast (Adler *et al.*, 1993) and pancreatic tumours (Bares *et al.*, 1994). These results suggest that the high uptake of FDG seems to be a general feature of a variety of cancers. Increased FDG uptake may reflect the high activity of hexokinase and glucose transport (Haberkorn *et al.*, 1994).

Calculation of the glucose metabolic rate using FDG based on Sokoloff's model has been applied to oncology PET, mostly to brain tumour studies (Di Chiro, 1987). However, this method requires arterial blood sampling and estimation of the lumped constant. Determination of the latter in individual tumours is impossible in humans. More simple evaluation methods without blood sampling, such as DUR or tumour–normal tissue ratio, have been recently introduced. The clinical value of these parameters has been demonstrated in several oncology studies. Zasadny and Wahl (1993) recently proposed the calibration of the FDG uptake with respect to body surface area or lean body weight, particularly in obese patients, instead of body weight. The

**Figure 3** The distribution of DUR of FDG in malignant (△) and benign (○) primary mediastinal tumours. Mean FDG uptake of malignant tumour is 7.15±2.27, benign tumour is 1.80±1.24.

**Table III** FDG uptake by thymic tumours

| *Histology (n)* | *DUR* |
|---|---|
| Thymic cancer (3) | 7.85±0.69 |
| Invasive thymoma (3) | 8.62±2.31* |
| Non-invasive thymoma (4) | 2.30±0.40 |
| Other benign tumours (8) | 1.56±1.46 |

Means±s.d. *$P<0.005$ compared with non-invasive thymoma and $P<0.001$ compared with other benign tumours. Not significant compared with thymic cancers (Student's *t* test).

FDG uptake of lung and head and neck tumours decreased significantly in the presence of hyperglycaemia (Lindholm *et al.*, 1993; Langen *et al.*, 1993). Our study did not include such obese patients as they have reported (BW 80–107 kg) and, furthermore, the blood glucose level was the same in patients with malignant and benign tumours. Therefore, technical errors in our PET measurements are unlikely.

A false-positive high FDG uptake was observed in sarcoidosis in the present study. Similar studies have recently described increased FDG uptake by enlarged lymph nodes (Lewis and Salama, 1994) and lung tissue (Brudin *et al.*, 1994) in sarcoidosis. Since sarcoid nodules consist of epidermoid cells that have, together with the macrophages, a common precursor cell, the monocyte, we believe that the high FDG uptake by the sarcoid tissue is a similar phenomenon to that by macrophages. This is supported by experimental demonstration of increased FDG uptake by macrophages (Kubota *et al.*, 1992).

The level of FDG uptake of tumours is related to the grade of malignancy in brain and soft tissue tumours (Di Chiro, 1987; Adler *et al.*, 1991). Furthermore, it has also been used as a prognostic indicator of malignancy in gliomas (Patronas *et al.*, 1985). The FDG uptake by the tumour also correlates with the cell density in grade 2 and 3 gliomas (Herholz *et al.*, 1993). Results of experimental studies indicate that the uptake of FDG is related to the number of viable cancer cells *in vitro* (Higashi *et al.*, 1993), and the amount of viable tissue *in vivo* (Kubota *et al.*, 1993). FDG uptake varies also with the histological differentiation of human abdominal tumours transplanted in nude mice (Yoshioka *et al.*, 1994). In this regard, recent studies from our laboratory indicate that FDG uptake by cancer cells is higher in $G_0/G_1$ and $G_2$ phases of the cell cycle compared with the S- and M-phases (Kubota *et al.*, 1994), and that tumour growth rates correlated with the FDG uptake of tumours (Kubota *et al.*, 1995). These results suggest that uptake of FDG by mediastinal tumours may represent a biological marker of the clinical behaviour of these tumours.

Thymomas are classified cytologically into benign or malignant. However, a proportion of the cytologically benign thymomas is locally invasive and has clinical features of malignancy. Thus, cytological classification may not be always prognostically useful (Lewis *et al.*, 1987). Our PET study demonstrated a high FDG uptake by thymic cancers and invasive thymomas. These results add support to the clinical practice that both thymic cancers and invasive thymomas should be treated as malignant tumours, while only non-invasive thymomas should be considered benign tumours (Lewis *et al.*, 1987). The classification of thymomas by PET in the present study agreed with the clinical rather than the cytological classification. However, owing to the small number of patients, a further confirmation of this observation is necessary.

Single-photon emission computerised tomography (SPECT) using $^{67}$Ga or $^{201}$Tl has been used widely for tumour imaging. Usefulness of $^{67}$Ga-SPECT for malignant lymphoma in mediastinum has been well established (Front *et al.*, 1991). However, the uptake of $^{67}$Ga is non-specific for malignant tumour (Tsan and Scheffel, 1986; Chandramouly *et al.*, 1989). The value of $^{67}$Ga for the differential diagnosis of mediastinal tumour seems not to be high. $^{201}$Tl-SPECT has been used recently for the diagnosis of lung and other tumours (Abdel-Dayem *et al.*, 1994). Detection of thymoma in patients of myasthenia gravis has been reported with $^{201}$Tl-SPECT. However, they cannot differentiate malignant tumour from benign thymoma (Tonami *et al.*, 1993).

In conclusion, PET, using FDG, seems to be useful in the evaluation of malignancy in primary mediastinal tumours. Both thymic cancer and invasive thymoma showed high FDG uptake, while non-invasive thymomas and other benign tumours showed low uptake. A high FDG uptake seems to reflect the invasiveness or malignant nature of thymic tumours.

**Acknowledgements**
The authors thank Mr Sugawara for photography, Mr Watanuki and Mr Seo for PET operation and Professor H Orihara, and the staff of the Cyclotron and Radioisotope Center, Tohoku University, for their assistance. We also thank Dr FG Issa (Word-Medex) for his assistance in reading and editing the manuscript. This work was supported by grants-in-aid (06454320, 06670899, 07274206) from the Ministry of Education, Science and Culture, Japan.

### References

ABDEL-DAYEM HM, SCOTT AM, MACAPINLAC HA, EL-GAZZAR AH AND LARSON SM. (1994). Role of $^{201}$Tl chloride and $^{99m}$Tc sestamibi in tumor imaging. In *Nuclear Medicine Annual 1994* Freeman LM (ed). Raven Press: New York, pp. 181–234.

ADLER LP, BLAIR HF, MAKLEY JT, WILLIAMS RP, JOYCE MJ, LEISURE G, AL-KAIST N AND MIRALDI F. (1991). Noninvasive grading of musculoskeletal tumors using PET. *J. Nucl. Med.*, **32**, 1508–1512.

ADLER LP, CROWE JP, AL-KAISI NK AND SUNSHINE JL. (1993). Evaluation of breast masses and axillary lymph nodes with [F-18]2-deoxy-2-fluoro-D-glucose PET. *Radiology*, **187**, 743–750.

ARONBERG DJ, GLAZER HS AND SAGEL SS. (1985). MRI and CT of the mediastinum comparisons, controversies, and pitfalls. *Radiol. Clin. North. Am.*, **23**, 439–448.

BARES R, KLEVER P, HAUTMANN S, HELLWIG D, FASS J, CREMERIUS U, SCHUMPELICK V, MITTERMAYER C AND BÜLL U. (1994). F-18 fluorodeoxyglucose PET in vivo evaluation of pancreatic glucose metabolism for detection of pancreatic cancer. *Radiology*, **192**, 79–86.

BROWN LR AND AUGHENBAUGH GL. (1991). Masses of the anterior mediastinum: CT and MR imaging. *Am. J. Roentgenol.*, **157**, 1171–1180.

BRUDIN LH, VALIND S-O, RHODES CG, PANTIN CF, SWEATMAN M, JONES T AND HUGHES JMB. (1994). Fluorine-18 deoxyglucose uptake in sarcoidosis measured with positron emission tomography. *Eur. J. Nucl. Med.*, **21**, 297–305.

CHANDRAMOULY BS, SCAGNELLI T AND BURGESS C. (1989). Uptake of gallium in the mediastinum. *Semin. Nucl. Med.*, **19**, 247–249.

CHEN J, WEISBROD GL AND HERMAN SJ. (1988). Computed tomography and pathologic correlations of thymic lesions. *J. Thorac. Imaging*, **3**, 61–65.

COHEN AJ, THOMPSON L, EDWARDS FH AND BELLAMY RF. (1991). Primary cysts and tumors of the mediastinum. *Ann. Thorac. Surg.*, **51**, 378–386.

DAVIS JR RD, OLDHAM JR HN AND SABISTON JR DC. (1987). Primary cysts and neoplasms of the mediastinum: recent changes in clinical presentation, methods of diagnosis, management, and results. *Ann. Thorac. Surg.*, **44**, 229–237.

DEWAN NA, GUPTA NC, REDEPENNING LS, PHALEN JJ AND FRICK MP. (1993). Diagnostic efficacy of FDG-PET imaging in solitary pulmonary nodules. *Chest*, **104**, 997–1002.

DI CHIRO G. (1987). Positron emission tomography using [$^{18}$F]fluorodeoxyglucose in brain tumors: a powerful diagnostic and prognostic tool. *Invest. Radiol.*, **22**, 360–371.

FRONT D, ISRAEL O AND BEN-HAIM S. (1991). The dilemma of a residual mass in treated lymphoma: The role of callium-67 scintigraphy. In *Nuclear Medicine Annual 1991*, Freeman LM (ed) Raven Press: New York. pp. 211–220.

GRAEBER GM, SHRIVER CD, ALBUS RA, BURTON NA, COLLINS GJ, LOUGH FC AND ZAJTCHUK R. (1986). The use of computed tomography in the evaluation of mediastinal masses. *J. Thorac. Cardiovasc. Surg.*, **91**, 662–666.

HABERKORN U, ZIEGLER SI, OBERDORFER F, TROJAN H, HAAG D, PESCHKE P, BERGER MR, ALTMANN A AND VAN KAICK G. (1994). FDG uptake, tumor proliferation and expression of glycolysis associated genes in animal tumor models. *Nucl. Med. Biol.*, **21**, 827–834.

HERHOLZ K, PIETRZYK U, VAGES J, SCHRÖDER R, HALBER M, TREUER H, STURM V AND HEISS W-D. (1993). Correlation of glucose consumption and tumor cell density in astrocytomas. *J. Neurosurg.*, **79**, 853–858.

HIGASHI K, CLAVO AC AND WAHL RL. (1993). Does FDG uptake measure proliferative activity of human cancer cells? In vivo comparison with DNA flow cytometry and tritiated thymidine uptake. *J. Nucl. Med.*, **34**, 414–419.

KUBOTA K, MATSUZAWA T, ITO M, FUJIWARA T, ABE Y, YOSHIOKA S, FUKUDA H, HATAZAWA J, IWATA R, WATANUKI S AND ITO T. (1985). Lung tumor imaging by positron emission tomography using C-11L-methionine. *J. Nucl. Med.*, **26**, 37–42.

KUBOTA K, MATSUZAWA T, FUJIWARA T, ITO M, HATAZAWA J, ISHIWATA K, IWATA R AND IDO T. (1990). Differential diagnosis of lung tumor with positron emission tomography: a prospective study. *J. Nucl. Med.*, **31**, 1927–1933.

KUBOTA R, YAMADA S, KUBOTA K, ISHIWATA K, TAMAHASHI N AND IDO T. (1992). Intratumoral distribution of fluorine-18-fluorodeoxyglucose in vivo: high accumulation in macrophages and granulation tissues studied by microautoradiography. *J. Nucl. Med.*, **33**, 1972–1980.

KUBOTA K, KUBOTA R AND YAMADA S. (1993). FDG accumulation in tumor tissue. *J. Nucl. Med.*, **34**, 419–421.

KUBOTA R, KUBOTA K, YAMADA S, TADA M, IDO T AND TAMAHASHI N. (1994). Active and passive mechanisms of [fluorine-18]fluorodeoxyglucose uptake by proliferating and prenecrotic cancer cells in vivo: a microautoradiographic study. *J. Nucl. Med.*, **35**, 1067–1075.

KUBOTA R, KUBOTA K, YAMADA S, TADA M, TAKAHASHI T, IWATA R AND TAMAHASHI N. (1995). Methionine uptake by tumor tissue: a microautoradiographic comparison with FDG. *J. Nucl. Med.*, **36**, 484–492.

LANGEN KJ, BRAUN U, KOPS R, HERZOG H, KUWERT T, NEBELING B AND FEINENDEGEN LE. (1993). The influence of plasma glucose levels on fluorine-18-fluorodeoxyglucose uptake in bronchial carcinomas. *J. Nucl. Med.*, **34**, 355–359.

LESKINEN-KALLIO S, NÅGREN K, LEHIKOINEN P, RUOTSALAINEN R AND JOENSUU H. (1991). Uptake of $^{11}$C-methionine in breast cancer studied by PET. An association with the size of S-phase fraction. *Br. J. Cancer*, **64**, 1121–1124.

LESKINEN-KALLIO S, NÅGREN K, LEHIKOINEN P, RUOTSALAINEN U, TERÄS M AND JOENSUU H. (1992). Carbon-11-methionine and PET is an effective method to image head and neck cancer. *J. Nucl. Med.*, **33**, 691–695.

LEWIS JE, WICK MR, SCHEITHAUER BW, BERNATZ PE AND TAYLOR WF. (1987). Thymoma: a clinicopathologic review. *Cancer*, **60**, 2727–2743.

LEWIS PJ AND SALAMA A. (1994). Uptake of fluorine-18-fluorodeoxyglucose in sarcoidosis. *J. Nucl. Med.*, **35**, 1647–1649.

LINDHOLM P, MINN H, LESKINEN-KALLIO S, BERGMAN J, RUOTSALAINEN U AND JOENSUU H. (1993). Influence of the blood glucose concentration on FDG uptake in cancer - a PET study. *J. Nucl. Med.*, **34**, 1–6.

LINK KM, SAMUELS LJ, REED JC, LOEHR SP AND LESKO NM. (1993). Magnetic resonance imaging of the mediastinum. *J. Thorac. Imaging*, **8**, 34–53.

MAZZIOTA JC, PHELPS ME, PLUMMER D AND KUHL DE. (1981). Quantitation in positron emission computed tomography: 5 Physical-anatomical effects. *J. Comput. Assist. Tomogr.*, **5**, 734–743.

MINN H, JOENSUU H, AHONEN A AND KLEMI P. (1988). Fluorodeoxyglucose imaging: a method to assess the proliferative activity of human cancer in vivo. *Cancer*, **61**, 1776–1781.

OKAZUMI S, ISONO K, ENOMOTO K, KIKUCHI T, OZAKI M, YAMAMOTO H, HAYASHI H, ASANO T AND RYU M. (1992). Evaluation of liver tumors using fluorine-18-fluorodeoxyglucose PET: characterisation of tumor and assessment of effect of treatment. *J. Nucl. Med.*, **33**, 333–339.

PATRONAS NJ, DI CHIRO G, KUFTA C, BAIRAMIAN D, KORNBLITH PL, SIMON R AND LARSON SM. (1985). Prediction of survival in glioma patients by means of positron emission tomography. *J. Neurosurg.*, **62**, 816–822.

PATZ EF, LOWE VJ, HOFFMAN JM, PAINE S, BURROWES P, COLEMAN RE AND GOODMAN PC. (1993). Focal pulmonary abnormalities: evaluation with F-18 fluorodeoxyglucose PET scanning. *Radiology*, **188**, 487–490.

REBNER M, GROSS BH, ROBERTSON JM, PENNES DR, SPIZARNY DL AND GLAZER GM. (1987). CT evaluation of mediastinal masses. *Comput. Radiol.*, **11**, 103–110.

RENDINA EA, VENUTA F, CERONI L, MARTELLI M, GUALDI G, CATERINO M AND RICCI C. (1988). Computed tomographic staging of anterior mediastinal neoplasms. *Thorax*, **43**, 441–445.

STRAUSS LG AND CONTI PS. (1991). The application of PET in clinical oncology. *J. Nucl. Med.*, **32**, 623–648.

TONAMI N, YOKOYAMA M, TAKI J, SHUKE N, MIYAUCHI T, MICHIGISHI T, ABURANO T AND HISADA K. (1993). Detection of thymic abnormality in myasthenia gravis with thallium-201 SPECT. *J. Nucl. Med.*, **34**, 223p.

TSAN MF AND SCHEFFEL U. (1986). Mechanism of gallium-67 accumulation in tumours. *J. Nucl. Med.*, **27**, 1215–1219.

WAHL RL, CODY RL, HUTCHINS GD AND MUDGETT EE. (1991). Primary and metastatic breast carcinoma: Initial clinical evaluation with PET with the radiolabelled glucose analogue 2-[F-18]-fluoro-2-deoxy-D-glucose. *Radiology*, **179**, 765–770.

YOSHIOKA T, TAKAHASHI H, OIKAWA H, MAEDA S, WAKUI A, WATANABE T, TEZUKA F, TAKAHASHI T, IDO T AND KANAMARU R. (1994). Accumulation of 2-deoxy-2-[$^{18}$F]fluoro-D-glucose in human cancer heterotransplanted in nude mice: comparison between histology and glycolytic status. *J. Nucl. Med.*, **35**, 97–103.

ZASADNY KR AND WAHL RL. (1993). Standardised uptake values of normal tissues at PET with 2-[fluorine-18]-fluoro-2-deoxy-D-glucose: variations with body weight and a method for correction. *Radiology*, **189**, 847–850.